# 923 Implementation of a Competency Based Orientation for Burn Nurses Using the ABA Burn Nurse Competencies

**DOI:** 10.1093/jbcr/iraf019.454

**Published:** 2025-04-01

**Authors:** Emily Werthman, Hall Bonnie, Julie Keenan

**Affiliations:** Johns Hopkins Burn Center; Johns Hopkins Burn Center; Johns Hopkins Burn Center

## Abstract

**Introduction:**

Prior to implementation, the burn center utilized a 12-week nursing orientation program for new nurses to the burn center. In addition to hospital-based orientation to EPIC and policies, this training included a competency-based orientation specific to burn nursing including pathophysiology, wound care, and resuscitation. Educational offerings following the orientation period were often ad hoc. Yearly trauma and burn education was often offered from other departments, instead of burn nursing. We identified continuing education for burn nurses as a gap and sought to implement a program to continue a competency-based education program for all burn nurses.

**Methods:**

Using the American Burn Association burn nurse competencies an education plan was developed for monthly, quarterly and yearly burn nurse competencies. The domains of initial management, physiologic support, wound management, pain agitation & delirium, nutritional support, psychosocial support, rehabilitation, discharge planning & aftercare, and end of life care were used to ensure offerings were well rounded and multi-disciplinary. To engage the multi-disciplinary team of the burn center, experts from across disciplines were used to present on their areas of expertise, i.e., the burn psychologist presented on the differences between acute stress response and post-traumatic stress disorder. Education is supported with institutional support in the form of 4 paid hours each week for continuing education, performance improvement work, or other nursing needs. As a result, we are able to compensate nurses for their time attending these trainings. Nursing management is also supportive of these efforts, requiring nurses to attend. Recordings of all offerings are made available to staff on the burn center’s intranet page.

**Results:**

Competency is assessed via a post-course assessment in conjunction with hands on training, where appropriate. For instance, in an offering that covers thermoregulation, nurses are required to complete a post-course written assessment as well as a hands on sign off on the use of fluid warmers, heat shields, and other warming measures.

**Conclusions:**

As this is a new program for continuing education we cannot compare it to previous efforts. However, year to year efforts have increased significantly. In the years prior to implementation of this program, there were few educational offerings given by burn nurses. In the last year, there were 12 monthly, 4 quarterly, and 1 annual update trainings provided.

**Applicability of Research to Practice:**

As the specialty of burn nursing continues to grow, we hope to expand to make our offerings publicly available. With burn nursing certification, the need for continuing education is increasing; we also aim to provide CEs for these trainings.

**Funding for the Study:**

N/A

